# Degloving intramuscular injuries of the semimembranosus and adductor longus muscles in adolescent soccer players

**DOI:** 10.1007/s00256-024-04757-6

**Published:** 2024-07-23

**Authors:** Marcelo Bordalo, Patricia Nunez de Aysa, Paulo Victor Partezani Helito, Mohamed Abdelatif Djadoun, Maria Lua Sampaio Gulde, Juan Manuel Alonso

**Affiliations:** Aspetar Orthopedic and Sports Medicine Hospital, Doha, Qatar

**Keywords:** Thigh, Semimembranosus, Adductor longus, Degloving injury, Intramuscular tendon, MRI, Ultrasound

## Abstract

Degloving muscle injury was described for the rectus femoris where the inner bipennate component is dissociated from its superficial unipennate component. The semimembranosus muscle displays a distinctive dual morphology, featuring both unipennate and bipennate muscle fibers. Nevertheless, this specific tear pattern has not been previously documented. Conversely, the adductor longus muscle showcases an elongated intramuscular tendon segment, indicating a multipennate morphology. We present two separate cases of previous undescribed degloving injuries of the semimembranosus and the adductor longus in teenage soccer players with MRI and ultrasound diagnosis, ultrasound-guided hematoma aspiration, and recovery timelines for return-to-play.

## Introduction

Hamstring and adductor strains are among the most common muscle injuries in sports that involve kicking, running, and jumping and are particularly prevalent in soccer [1, 2]. Particularly in professional football, muscle injuries are an important cause of time-loss injuries, with hamstring and adductor injuries accounting for more than 50% of muscle injuries [2]. Among hamstring injuries, semimembranosus (SM) strains are less prevalent than injuries of the biceps femoris and semitendinosis (ST). Moreover, adductor longus tears are common in football and are the main causes of groin injuries in athletes [3].

Understanding specific patterns of muscle injuries is crucial, as different injuries can have potentially different prognoses and necessitate varied patient care approaches. This principle is exemplified in a unique type of muscle tear previously described by Kassarjian et al., which specifically occurs in the indirect myotendinous complex of the rectus femoris [4]. This injury, known as an intramuscular degloving injury, accounted for 9% of all rectus femoris injuries in their series. Characterized by the separation of the inner bipennate muscle belly from the superficial unipennate muscle, with retraction of the inner muscle belly, this injury presents a distinct clinical challenge. Although degloving injuries are typically intramuscular tears that do not involve the myotendinous or intratendinous region, they are clinically significant due to their propensity to impair mobility and function [4].

We report two cases of previously undescribed degloving injuries to the SM and adductor longus muscles in teenage football players. We discuss clinical and imaging aspects, including ultrasound (US) and magnetic resonance imaging (MRI) findings, treatment, and follow-up until return to play.

## Case reports

Informed consent was obtained from the subjects’ parents.

### Case 1

A 14-year-old male football player, mid-fielder, right leg dominant, experienced acute pain (visual analog scale (VAS) score, 7/10) in his posterior right thigh while kicking the ball in a football match 3 days prior. Physical examination revealed a normal gait, no thigh swelling or bruising, mild tenderness on palpation of the proximal aspect of the medial hamstrings, and pain on resistance testing of the hamstrings.

**Fig. 1:**
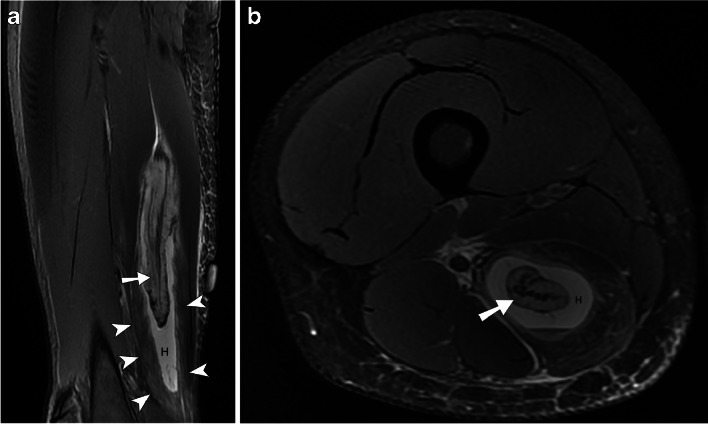
Degloving injury of the semimembranosus muscle. Sagittal (**A**) and axial (**B**) T2 weighted images with fat saturation depict a circumferential muscle injury around the proximal myotendinous junction (arrow) with separation of inner muscle fibers from the peripheral semimembranosus muscle fibers (arrowheads) and interposed hematoma (H)

**Fig. 2:**
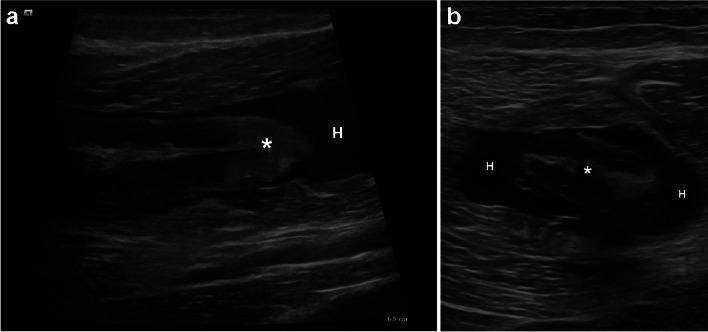
High-frequency ultrasound scan with longitudinal (**A**) and transverse (**B**) images depicting the degloving tear, with the inner muscle separated from the peripheral muscle fibers by interposed hematoma (H)

**Fig. 3:**
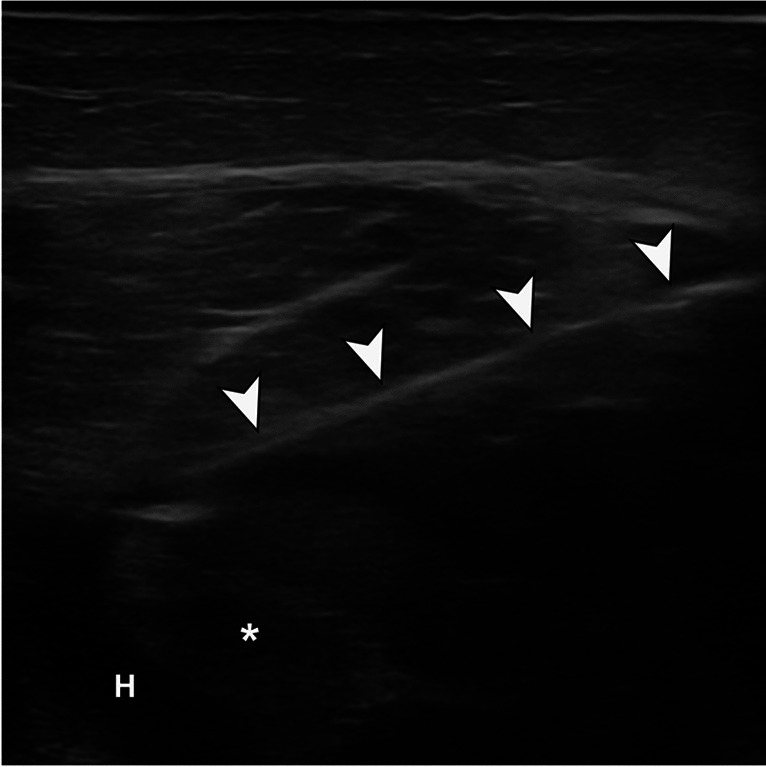
Ultrasound-guided aspiration of the hematoma with an in-plane transversal and posteromedial approach. Needle (arrowheads) has the distal tip at the hematoma (H). Approximately 80 ml of hematic fluid was aspirated from the injury. Asterisk: degloved inner muscle fibers

With a clinical suspicion of a hamstring muscle injury, an MRI was performed 4 days after the injury and revealed partial muscle detachment from the proximal SM inner muscle belly and myotendinous junction and from the outer muscle belly and fascia, with an interposing hematoma of approximately 80 ml. The lesion measured 20.6 × 3.9 × 2.5 cm, with 5.2 cm of retraction (Fig. 1). Because of the large volume, we decided to aspirate the hematoma at an early stage to facilitate early healing. US-guided aspiration of 78 ml of hematic fluid was performed on the same day (Figs. 2 and 3).

The patient underwent a six-stage hamstring rehabilitation protocol and was fit to return to play 53 days after the injury.

### Case 2

**Fig. 4:**
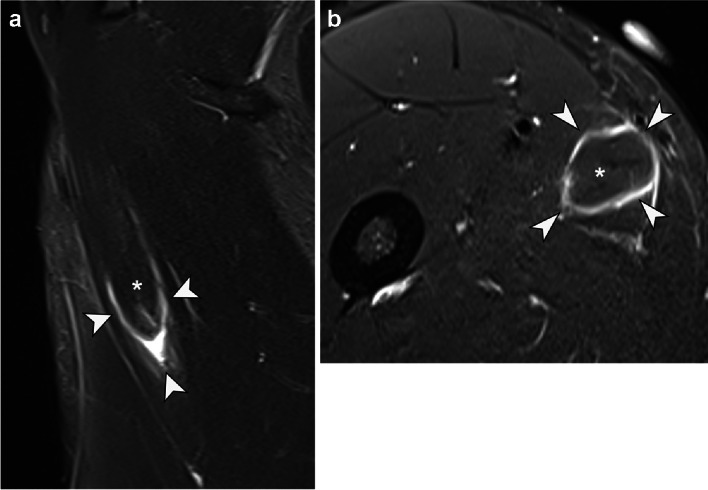
Degloving injury of the adductor longus muscle. Axial (**A**) and sagittal (**B**) T2 weighted images with fat saturation depict a muscle injury in the adductor longus muscle, around the distal aspect of the proximal myotendinous junction (asterisk), with complete separation of the myotendinous junction from the peripheral muscle fibers and fascia (arrowheads). There is a small amount of fluid surrounding the tear

A 14-year-old male football player, striker, right leg dominant, presented sudden pain in the right groin during a training session 2 weeks prior, while he was kicking long balls (VAS score, 8/10). Physical examination revealed adductor-related groin pain. MRI revealed partial detachment of the proximal adductor longus inner muscle belly from the outer muscle belly, with a small associated fluid collection (9 ml), consistent with a degloving injury. The lesion measured 7.0 × 2.7 × 3.5 cm, with 1.7 cm of retraction (Fig. 4). The decision was made not to aspirate, given the limited volume of the hematoma. The player started a specific four-phase rehabilitation program for acute adductor injury and was able to return to play 35 days after the injury.

## Discussion

Muscle architecture plays a crucial role in determining the specific location and characteristics of injuries. A degloving muscle tear is secondary to a muscle-within-a-muscle anatomy, which makes the muscle susceptible to separation of its unipennate and bipennate portions. Specifically, the bipennate component consists of fibers originating from both sides of the tendon, a feature commonly observed in muscles with an intramuscular central tendon. In contrast, the unipennate component involves muscle fibers that originate from a single side of the tendon, situated alongside the muscle, occasionally merging with the superficial muscle aponeurosis. This unique type of tear was originally described for the rectus femoris muscle, which possesses an intramuscular tendon stemming from the indirect tendinous component and a superficial tendon arising from the direct component and blending with the superficial aponeurosis [4].

Degloving injuries have been described only in the rectus femoris muscle because of its classical unipennate and bipennate muscle architecture. Nevertheless, it is noteworthy that other muscles exhibit an intramuscular tendon component that is susceptible to analogous degloving injury patterns. This is, to our knowledge, the first description of intramuscular degloving injuries extending beyond the rectus femoris muscle.

**Fig. 5:**
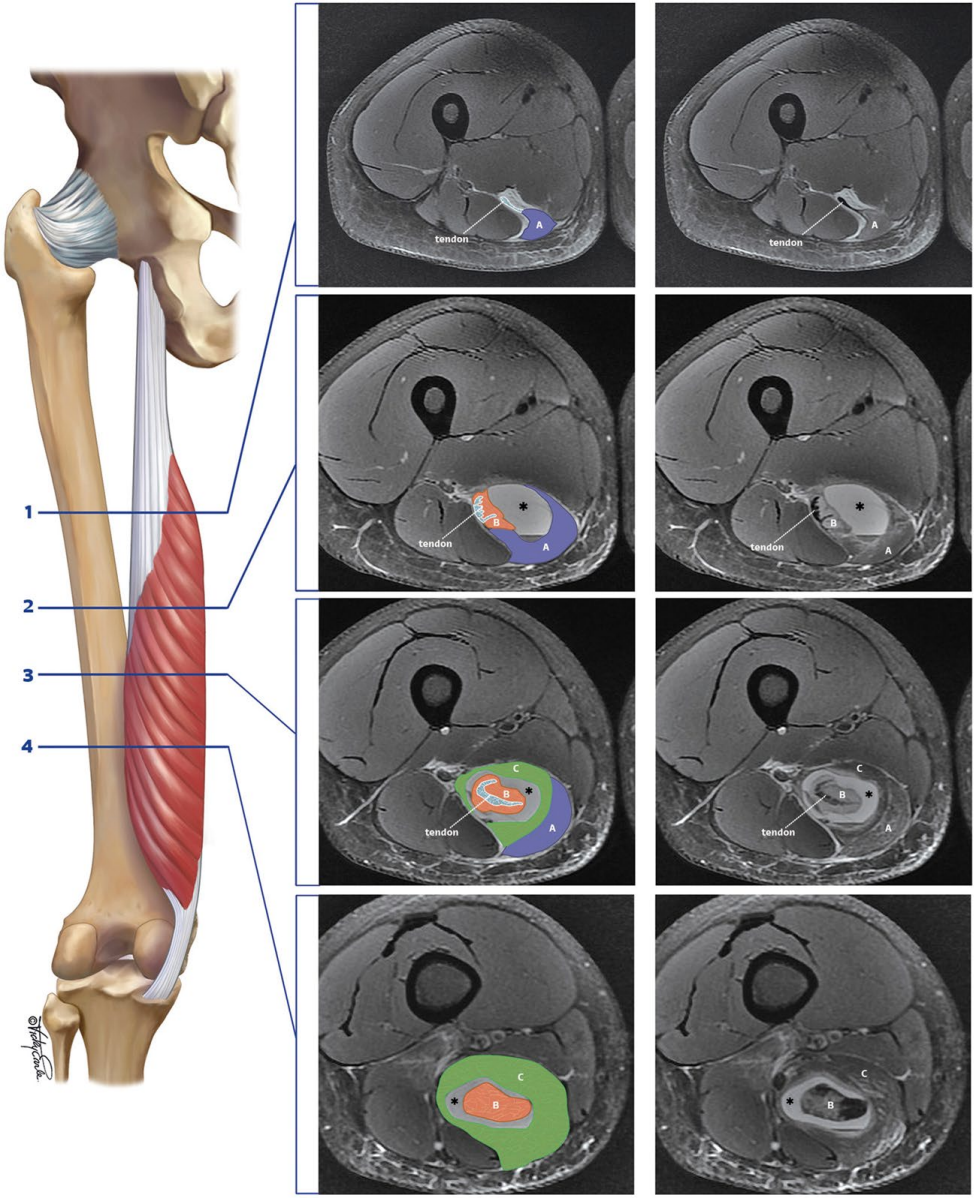
Semimembranosus pennation architecture. Illustration of the semimembranosus anatomy correlated with the axial MR images at four levels. The semimembranosus fibers define three anatomical regions based on pennation and fiber orientation [5, 6]. Region A is characterized as unipennate and is located more proximally, originating from the proximal tendon (see levels 1, 2, and 3); region B exhibits a bipennate structure, with fibers originating from the proximal tendon and encircling the intramuscular tendinous component (see levels 2, 3, and 4); and region C is unipennate, envelops the bipennate region B, and attaches to the distal tendon (see levels 3 and 4). The hematoma is surrounding region B (*)

The SM has a muscle-in-muscle configuration, consisting of two unipennate regions and one bipennate region [5]. Balius et al. illustrated the distribution of the three distinct regions of the SM muscle, predicated on the anatomical arrangement of tendons and muscle units, on axial T1-weighted MRI [6]. In our cases, we noted a dissociation within the SM muscle, characterized by a separation between the inner muscle belly, which encompasses the proximal central tendon, and the surrounding outer muscle component. The inner muscle component, originating from the lateral surface of the proximal tendon and encompassing the central tendon, aligns with the bipennate region (Fig. 5).

**Fig. 6:**
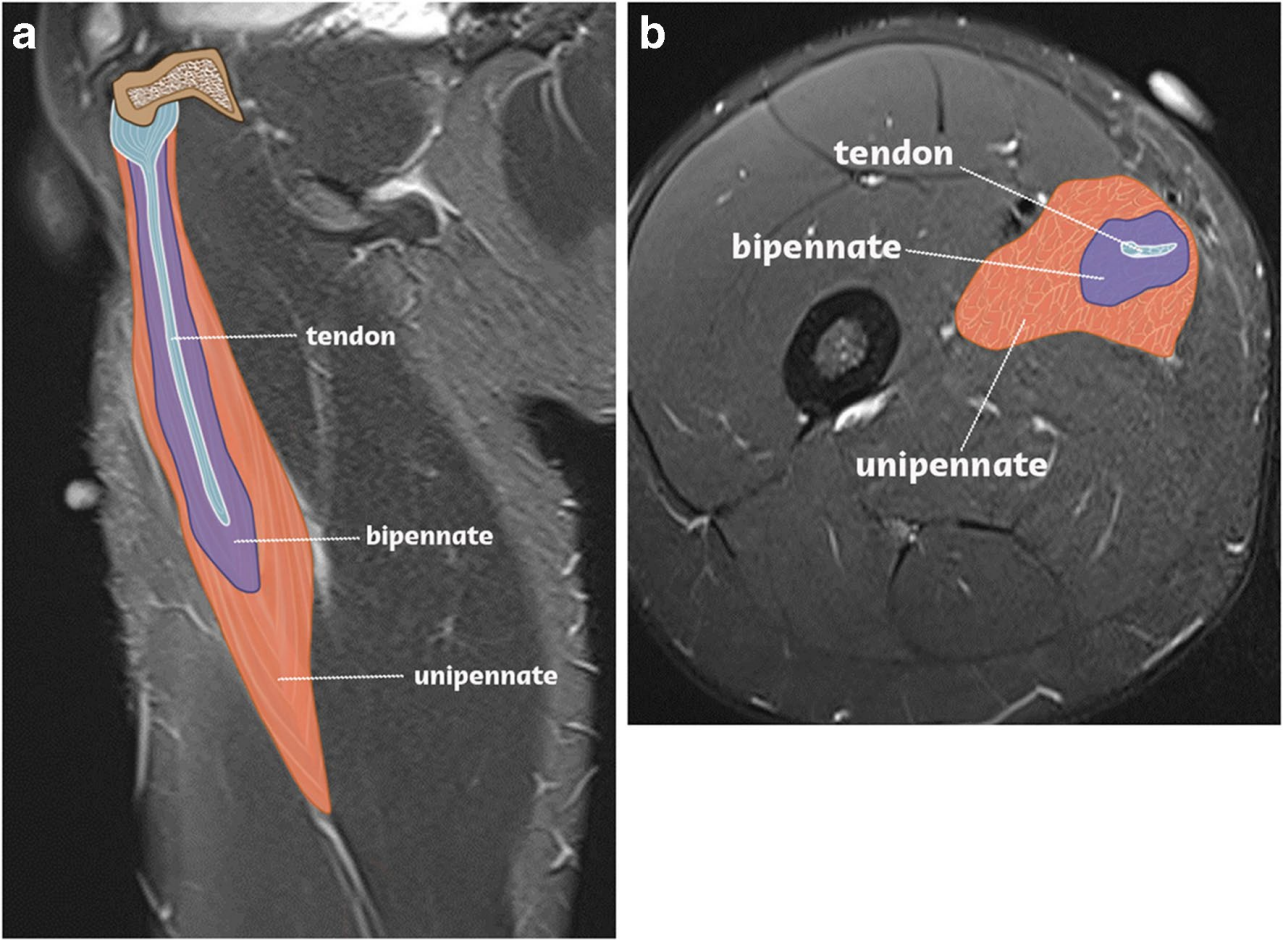
Adductor longus pennation anatomy. Sagittal (**A**) and axial (**B**) MR images of the adductor longus correlated with an illustration of the pennation anatomy. The superficial unipennate component envelops the inner bipennate component, which in turn encircles the intramuscular tendon

A pennate muscle architecture has not been reported for the adductor longus. Nevertheless, it is noteworthy that a deep intramuscular tendon forms an elongated myotendinous junction within the adductor longus muscle [7]. The occurrence of an intramuscular degloving injury surrounding an intramuscular tendon suggests the presence of a muscle-in-muscle configuration within the adductor longus (Fig. 6).

Regarding the mechanism of injury, both of our cases were similar—teenage male soccer players with stretch-related mechanisms while kicking the ball, with an eccentric muscle contraction accompanied by hyperflexion of the hip and extension of the ipsilateral knee [8]. The resemblance observed in the muscle injury pattern across distinct muscle groups, along with evidence from prior literature [4], posits that the stretching-type injury incurred during the act of kicking a ball aligns more closely with the degloving subtype of muscle injury.

The return-to-play durations noted in our case studies closely correspond to the documented average of 38.7 days for rectus femoris degloving injuries, which parallels the timeframe for intratendinous type tears (35–130 days) [9]. These durations exceed those associated with purely myofascial or myotendinous tears (12–34 days) [10]. It has been proposed to categorize this injury as an intramuscular tear rather than a myotendinous tear, owing to the absence of direct involvement of the myotendinous junction, superficial aponeurosis, or central tendon in degloving injuries. However, a rigorous evaluation of return-to-play intervals and distinct clinical manifestations suggest that degloving type muscle injuries may be a prognostic radiological finding comparable to intratendinous type injuries, as opposed to myofascial, intramuscular, or myotendinous tears.

Degloving injuries typically involve varying quantities of hematoma accumulation. Presently, the literature lacks definitive guidance regarding the indications for and optimal timing of hematoma aspiration. Furthermore, the potential advantages of aspiration in terms of muscle recovery and facilitating return to play in athletes remain to be elucidated [11]. Aspiration may be considered in instances where there is an intramuscular fluid collection accompanied by severe pain. Theoretically, aspiration could facilitate early healing and reduce the return-to-play time in athletes. However, empirical evidence supporting these hypotheses is currently lacking, and further investigation is warranted [12, 13]. Although the sonographic presentation of a hematoma is not inherently correlated with its age, it is notable that hypoechoic hematomas are generally more amenable to aspiration than hyperechoic or complex hematomas [13].

In summary, we present two rare cases of intramuscular degloving injuries of the SM and adductor longus in teenage football players, as documented by MRI and US.
